# The Relation between Hearing Loss and Smoking among Workers Exposed to Noise, Using Linear Mixed Models

**DOI:** 10.22038/ijorl.2019.37555.2229

**Published:** 2020-01

**Authors:** Fatemeh Khaldari, Narges Khanjani, Abbas Bahrampour, Mohammad Reza Ghotbi Ravandi, Ali Asghar Arabi Mianroodi

**Affiliations:** 1 *Department of Biostatistics and Epidemiology, Faculty of Health, Kerman University of Medical Sciences, Kerman, Iran.*; 2 *Neurology Research Center, * *Kerman University of Medical Sciences* *, Kerman, Iran.*; 3 *Department of Occupational Health, Faculty of Health, Kerman University of Medical Sciences, Kerman, Iran.*; 4 *Department of Otorhinolaryngology, Faculty of Medical, Kerman University of Medical Sciences, Kerman, Iran.*

**Keywords:** Hearing Loss, Noise-Induced, Linear Models, Longitudinal Studies

## Abstract

**Introduction:**

Noise is one of the most common and harmful physical factors in the working environment and has physical and psychological effects on individuals. In this study, the audiometry results of industrial workers were modeled and the effect of noise and other factors on hearing loss was examined.

**Materials and Methods:**

This was a longitudinal study based on the records of workers who had worked over 10 years in the industry and had recorded audiometries since their employment. Data was analyzed through linear mixed models.

**Results:**

During each year of noise exposure, hearing loss was 1.9 db at 4000 Hz; 0.059 in low frequencies and 0.62 db in high frequencies. At 8000 Hz the effect of the age at employment on hearing loss was significant (P=0.014). At low frequencies the interaction of smoking and age at employment was significantly related to hearing loss (P˂0.001).

**Conclusion:**

This study showed that despite acquaintance with safety measures, workers still face hearing loss in industry and employers should put workers under more surveillance for using protective gear. Smoking might be another risk factor for hearing loss.

## Introduction

Nowadays, due to population growth and establishment of large-scale industries; the use of machinery, equipment, processes and chemicals is inevitable. Industrialization introduces various hazards to the workforce and makes the workers face many harmful factors. One of the occupational hazards triggered by technological progress in industrial fields and the extensive use of equipment and machinery is the unpleasant acoustic turmoil, called noise (1,2).

Among all the physical occupational pollutants, noise has the most emission in many industries. Noise not only causes disease, but can also be an annoyance, cause anger, and interfere with conversations. It can also prevent hearing the warning sounds, cause accidents, and reduce production (1). Recent studies have also shown that occupational noise exposure can damage DNA (3), increase stress hormones, especially norepinephrine (4), worsen sleep quality and increase the production of free radicals (5,6).

Noise is the most common harmful physical factor in work environments in the world. Approximately, 600 million workers are exposed to noise in the work environment (7).

It is estimated that in the United States about 22.4 million workers are exposed to workplace noise and more than 100 million people are exposed to noise from non-occupational situations such as traffic, use of personal devices, and other sound sources (8).

Hearing loss is one of the main occupational diseases in Europe. According to the estimates by the Occupational Safety and Health Organization, 17% of workers in the manufacturing sector are hearing impaired (9).

Noise pollution is not confined to countries that have advanced technology. In many developing countries, noise pollution maybe even more severe; and the proportion of temporary or permanent hearing loss is higher (9). Several studies conducted in Iran have shown high noise exposure in industries(10-12).

There are no accurate statistics regarding the rate of exposure to industrial noise in Iran, but one can imagine that the extent of the problem in Iran is significant. Naturally, every study examining the effect of noise and factors affecting hearing loss in workers is very important in Iran (13). Estimates say that about 2 million workers are exposed to hazardous noise in Iran (14).

Accordingly, nowadays, to increase productivity and reduce the effects of noise on the human body, noise research has attracted serious attention in many countries (15). Due to growing industrialization in societies and exposure to a lot of noise, acquired sensorineural hearing loss is a global problem and preventing it is highly crucial. The most common cause of occupational hearing loss is long-term exposure to noise above 85dB in the workplace (16).

An important point is that hearing loss induced by noise is easily prevented, but it is irreversible after its incidence (17). Typically, in the early years of work, hearing loss in the frequency range of 3000 to 6000 Hz becomes apparent, with the maximum loss at 4000 Hz. With continued exposure to noise, hearing loss increases and includes other frequency levels as well (1,18). For people who work in places with a noise intensity of greater than 85 dB, performing periodical audiometric tests is highly important to identify people with cochlear dysfunction in the early stages and keep them away from sound sources (18).

One of the oldest, simplest, and yet the most reliable audiological tests is pure sound audiometry or PTA. Audiometry is a qualitative and quantitative test of hearing. The device works by creating pure frequency sound signals with different intensities which can help determine the individual’s threshold of hearing at certain frequencies. In this study, the hearing threshold changes have been studied, based on the available recorded periodic audiometries in workers exposed to noise.

Although the relation between noise and hearing loss and the necessity of using protective equipment is obvious, but hearing loss is still happening among factory workers in Iran and more research about preventing hearing loss is needed.

The statistical models used in this study have not been used before in hearing loss, and can show new dimensions of hearing loss research.

## Materials and Methods

The current study is a longitudinal study with repeated measures in which several measurements are done on a particular person at different times. The primary objective in a longitudinal study is evaluating a response variable's changes over time and factors influencing it. There are two important groups of changes in longitudinal studies; one is an individual’s changes over time and the other is variations between individuals. In longitudinal studies, the data have a hierarchical structure at two levels. Repeated measurements form the first level units, and the individuals form the second level units. In such data, observations are not independent, hence appropriate models that take care of the dependencies between observations should be used. One of the most widely used statistical methods for inferring repeated measures is the Multivariate Mixed Analysis of longitudinal data. These models consider the random effects in modelling, model individual changes and render more accurate results. They are called mixed models, because they are a combination of random effects and fixed effects in one model (19).

 The general formula for a mixed model is:

yi= Xiβ + Zibi+ εi

bi∼Nq(0,Ψ)

εi∼Nni(0,σ2Λi)

where yi is the ni×1 response vector for observations in the ith group, Xi is the ni×p model matrix for the fixed effects for observations in group i, β is the p ×1 vector of fixed-effect coefficients, Zi is the ni×q model matrix for the random effects for observations in group i, bi is the q ×1 vector of random-effect coefficients for group i, εi is the ni×1 vector of errors for observations in group i, Ψ is the q ×q covariance matrix for the random effects, σ2Λi is the ni×ni covariance matrix for the errors in group i (20).

The current research is a longitudinal study, and has included the audiometries of workers at several time points, and data analysis has been done by Linear Mixed Models.

The study was performed on workers of the Shahid Bahonar Ancillary Copper Industries, Kerman, Iran; exposed to noise above 85dB. The Shahid Bahonar Ancillary Copper Industries Co. is the largest supplier of copper products and its alloys. The complex has three main melting and casting, extrusion, and rolling manufactories. From 800 workers, those who were exposed to noise above 85dB entered the study. Workers under 10 years of experience, workers with previous jobs or second jobs exposed to noise, workers with hearing problems from before employment, long-term use of ototoxic drugs, suffering from acute hearing injuries, and workers with tympanic membrane rupture were excluded. However, if the worker had the so-called problems and more than 10 years of experience prior to the incidence of the disorder, their previous audiometries were used. After reviewing the records and applying the exclusion criteria, 273 workers were indicated as eligible to enter the analysis. From these 273 workers, one worker had only one audiometry record and was excluded from the analysis.

The random slope model and random intercept model are multilevel regression models. In our analysis, the individuals were considered as groups. There was also the possibility to consider the ear side (right or left) as nested within the individual, because each person had two ear audiometries. The ear side (right or left) analysis was done, and since there was no significant difference, the individuals were considered as groups and each group contained one person measured twice.

The random intercept model is a model that allows random intercepts for different groups which is here equivalent to different people. That is, in this model, rather than assuming that all workers’ hearing thresholds were on average the same at the onset and that the thresholds increase with increase of work experience with the same slope (linear model), different people were let to have different hearing thresholds at the beginning which increased with increasing experience with a constant slope (i.e., individual differences were considered in the initial auditory thresholds, but they were not considered regarding the increase of hearing threshold affected by work experience).

The random slope model allows random slopes of the regression line for different groups which are equivalent to different people. That is, rather than considering the average hearing threshold caused by increase in work experience as the regression line slope for all individuals, it takes into account individual differences in increasing hearing threshold affected by increase in work experience. It is assumed that all individuals have an average initial hearing threshold; however, the impact of increase in work experience is different in hearing thresholds of different individuals.

Therefore, the best model allows different baseline hearing thresholds and different line slopes for different workers, and this is the Random Slopes and Intercepts Model. Analysis of variance and how much the model justified the data variances, and comparing the Maximum Likelihood of models, showed that this model explained the data better.

Regarding the fact that the hearing loss caused by noise occurring in closed buildings where workers are constantly moving is bilateral, the analysis was performed via the simplest multilevel model and then by entering an additional level for each ear. This analysis showed that the difference between the left and right ear was not statistically significant. So the audiometry tests from the left and right ears were analyzed together.

After analysis at different frequencies, the hearing threshold at low frequencies (HTL-L) which is the mean hearing threshold in frequencies 500, 1000, and 2000 Hz, and the hearing thresholds at high frequencies (HTL-H) which is the mean hearing threshold at 4000 and 8000Hz were also studied.

It would have been better if the hearing threshold in high frequencies were calculated based on the average hearing thresholds at 3000, 4000, and 6000 Hz; which are mostly damaged by exposure to noise. But, because no measurements were done in the frequencies of 3000 and 6000, the frequencies of 4000 and 8000 Hz were considered instead. Data analysis was performed by Stata 13.

## Results

Eligible workers who participated in the study were 272 persons, and their average work experience was 14.5±2.27 (mean±SD) years. The average age of the subjects in the first audiometry was 24.97± 3.22 years, and the mean age at the last audiometry was 37.28±5.49 years, and 90% of the workers were under 43 years of age. Between 3 to 7 audiometric measurements were available for each worker. The average number of audiometries was 4.1.

One hundred and seven workers (i.e. 39.34% of the workers) were smokers and 245 of them (90%) had normal blood pressure (i.e. ≤140/90 mmHg). Descriptive data of other independent variables are in (Table.1), and descriptive data of the dependent variables in (Table.2).

**Table 1:** Descriptive data of workers independent variables

	Mean	Standard deviation	25 percentile	Median	75 percentile
Systolic blood pressure	125.6	0.93	115	120	135
Diastolic blood pressure	7.66	0.59	70	85	85
BMI	24.99	3.72	22.86	24.81	27.10
Initial hearing threshold at 250 Hz	12.05	4.15	10	10	15
Initial hearing threshold at 500 Hz	11.60	3.99	10	10	15
Initial hearing threshold at 1000 Hz	10.79	3.85	10	10	10
Initial hearing threshold at 2000 Hz	10.59	3.97	10	10	10
Initial hearing threshold at 4000 Hz	12.25	4.71	10	10	15
Initial hearing threshold at 8000 Hz	12.90	4.58	10	10	15

**Table 2:** Descriptive data of workers dependent variables

The hearing threshold at different frequencies	No.	Mean	Standard deviation	25 percentile	Median	75 percentile
250 Hz	2240	19.88	6.74	15	20	20
500 Hz	2240	18.53	7.11	15	20	20
1000 Hz	2240	18.19	6.18	15	20	20
2000 Hz	2240	18.58	7.67	15	20	20
4000 Hz	2240	23.64	9.65	15	20	30
8000 Hz	2240	24.56	10.07	20	20	30
Low frequencies	2240	19.75	6.65	16.67	20	25
High frequencies	2240	25.80	10.14	20	25	30

Workers participating in the study all had more than 10 years of work experience, and all workers underwent evaluation from the beginning of employment and during their work years. 

**Fig 1:**
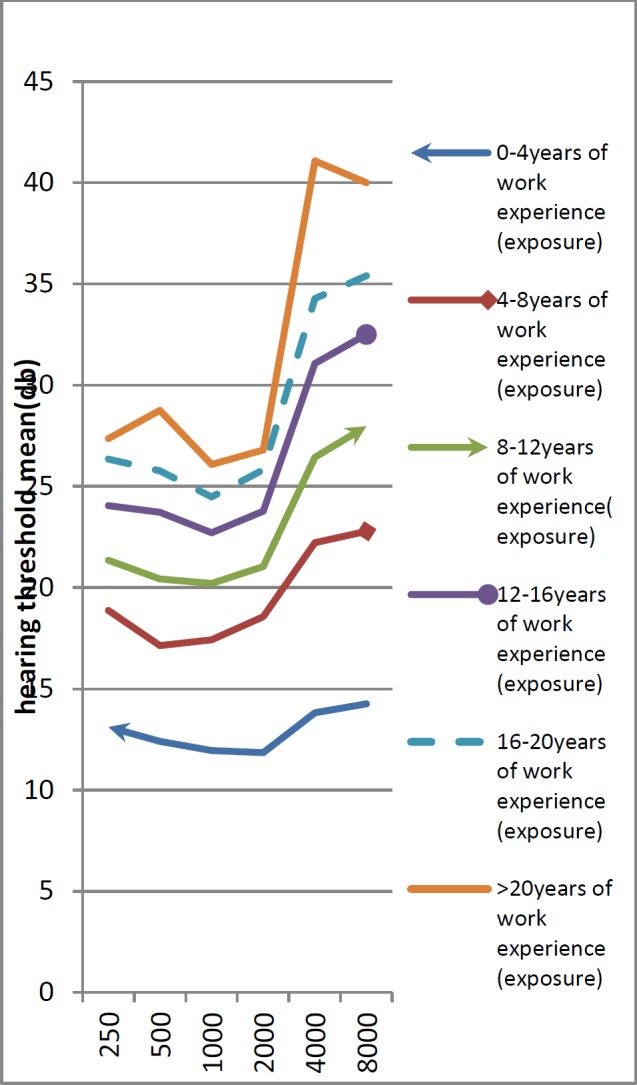
The average hearing threshold at different periods of work experience (exposure)

In Figure 1, the variations of the mean hearing threshold at different frequencies are depicted for workers exposed to noise during 4 year periods in terms of work experience which started from the beginning of employment. It is observed that when work experience (exposure) increases, the hearing threshold also increases, and the greatest loss occurred at 4000 Hz.

**Fig 2:**
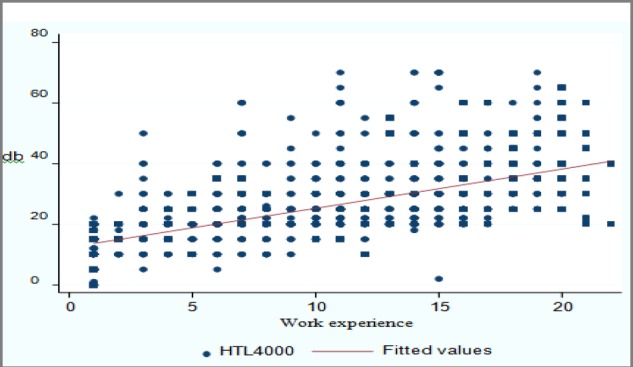
The relation between hearing threshold at 4000 Hz and work experience

Figure 2 shows the linear crude relation between job experience and hearing loss in 4000 Hz. 

**Fig 3:**
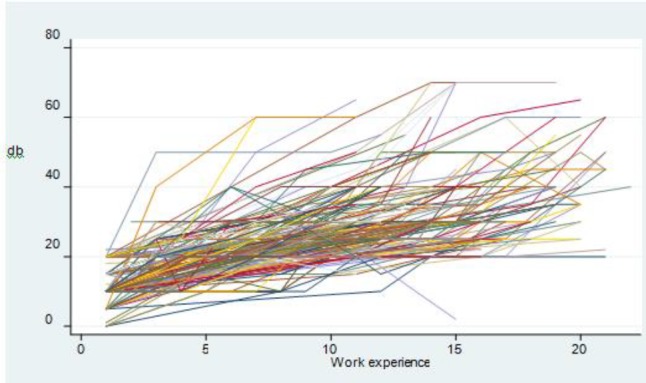
Hearing threshold variations at 4000 Hz for each person in relation to work experience (Spaghetti Graph)

As it can be seen hearing loss increases with increased job experience. Figure 3 shows Spaghetti graphs of the relation between job experience and hearing loss at 4000Hz for each individual worker.

Data analyses based on the Linear Mixed Model with both the constant and random effects are shown in Table 3. Table 3 shows that as job experience and the baseline threshold increased, hearing loss increased as well. The interaction between job experience and baseline hearing threshold was negative and showed as the baseline hearing threshold of the workers increased, the slope of hearing loss decreased with increased job experience.

The interaction between years of work experience and age at start of employment was positive and showed that as age at start of employment increased, the slope of hearing loss increased with increase in work experience. The interaction between work experience and smoking was negative in some frequencies and showed that as job experience increased the slope of hearing loss with smoking decreased. Table 4 shows the results of linear mixed model analysis (both fixed and random effects) for hearing loss at low and high frequencies. The interaction between age at employment and smoking was negative and shows that as age at employment decreases, the slope of hearing loss with smoking increases.

**Table 3:** The results of linear mixed model analysis for hearing loss at different frequencies

Hearing threshold	250 Hz (p-value)	500 Hz (p-value)	1000 Hz (p-value)	2000 Hz (p-value)	4000 Hz (p-value)	8000 Hz (p-value)
Variables						
Work experience (years)	1.83 (˂0.001)	1.66 (˂0.001)	1.32 (˂0.001)	1.20 (˂0.001)	1.90 (˂0.001)	1.32 (˂0.001)
Initial hearing threshold	0.7368 (˂0.001)	0.7711 (˂0.001)	0.7308 (˂0.001)	0.6848 (˂0.001)	0.6397 (˂0.001)	0.6176 (˂0.001)
Age at employment	-	-	-0.4136 (0.340)	-0.0841 (0.085)	-	-0.1801 (˂0.001)
BMI	0.0661 (0.257)	-	0.5393 (0.040)	-	-	-
Systolic blood pressure	-	-	-0.2418 (0.033)	-	-	-
Diastolic blood pressure	-	-	-2.64 (0.042)	-	-	-
Smoking	0.2457 (0.452)	-	0.1918 (0.519)	-	-	-0.0079 (0.986)
Basic hearing threshold × work experience	-0.0598 (˂0.001)	-0.0604 (˂0.001)	-0.0553 (˂0.001)	-0.0562 (˂0.001)	-0.0347 (˂0.001)	-0.0434 (˂0.001)
work experience × age at employment	-	-	0.0134 (0.055)	0.0174 (0.05)	-	0.0456 (˂0.001)
work experience × smoking	-0.1183 (0.033)		-0.0970 (0.040)	-	-	-0.2024 (0.04)
Diastolic blood pressure × Age at employment	-	-	0.1050 (0.039)	-	-	-
Age at employment × BMI	-0.0021 (0.175)	-	-	-	-	-
Subject random intercept var (SD)	0.0980 (0.3283)	0.2336 (0.4833)	0.1826 (0.4274)	0.0415 (0.2038)	0.0547 (0.2338)	0.0812 (0.2849)
Subject random slope var (SD)	0.1077 (0.3130)	0.1331 (0.3649)	0.0734 (0.2709)	0.1487 (0.3856)	0.3363 (0.5799)	0.4373 (0.6613)
Residual var (SD)	15.52 (3.94)	15.84 (3.98)	12.47 (3.53)	13.74 (3.70)	24.93 (4.99)	30.96 (5.56)

**Table 4:** The results of linear mixed model analysis for hearing loss at low and high frequencies

	HTL-L (p-value)	HTL-H (p-value)
Work experience	0.0597 (0.026)	0.6261(˂0.001)
Initial hearing threshold	0.0309 (0.047)	0.2132 (˂0.001)
Age at employment	- 0.4009 (˂0.001)	- 0.4780 (˂0.001)
Smoking	- 2.24 (˂0.001)	-
Diastolic blood pressure	1.30 (˂0.001)	-
Initial hearing threshold × Age at employment	0.0393 (˂0.001)	0.0361(˂0.001)
Age at employment × smoking	0.0925 (˂0.001)	-
Diastolic blood pressure×Age	- 0.0541 (˂0.001)	-
Initial hearing threshold × work experience	- 0.0040(0.046)	-
Subject random intercept var (SD)	0.9208 (0.9595)	0.0862 (0.2937)
Subject random slope var (SD)	0.0185 (0.1362)	0.1926 (0.4438)
Residual var (SD)	0.1598 (0.3998)	14.38 (3.79)
Log likelihood	-1810.043	-6475.51

## Discussion

This study was a longitudinal research conducted to evaluate the effects of noise on workers hearing, according to sequential audiometries done during more than 10 years of work. Most studies done in this area were cross sectional studies, but this study had a different approach. The main purpose of this study was to describe the changes in hearing threshold levels in a group of workers exposed to noise above 85 dB, for 8 hours a day. Considering the fact that workers in different parts were constantly moving and their workplace was a closed space, bilateral and symmetrical hearing loss was observed in both ears.

The relation between hearing loss and aging has been confirmed in other studies (7,21). Age increase does effect hearing loss (22); but, more than 80% of people exposed to noise in this study were less than 40 years old at the time of the study. Researches think the start of age-induced hearing loss is after the age of 40 and another study estimated the average age at which age-induced hearing loss starts is 51.5 years in men and 51.2 in women (23,24). Therefore, the main cause of hearing loss in the recent study was probably accumulated noise exposure which increased with increase in work experience.

In the industry evaluated in this study, the use of engineering control measures to reduce noise intensity was not fully possible in different parts of the plant. Thus, personal hearing protection devices, such as airplugs and airmuffs, had be to used, but their use was very irregular in these workers.

Permanent hearing loss due to continued exposure to noise over 85 dB is primarily seen at 4000 Hz and if exposure continues, other frequencies will get involved later, as well. Noise-induced hearing loss is usually bilateral and symmetric in both ears, but because of working conditions, it can be more severe in one ear (25). In Rogha et al.'s study conducted in textile workers; there was no significant difference in hearing loss between their two ears (26).

In a study conducted by Abedi et al. on the hearing loss of airport staff, there were no significant differences between right or left ear hearing loss, confirming the fact that the harmful effects of noise on the hearing system is bilateral and symmetric (2). Also in the current study, no significant differences were observed regarding noise-induced hearing loss in both ears.

But according to a study conducted on the frequency of hearing loss among drivers of heavy vehicles by Berjis et al., left ear hearing loss was significantly higher than the right ear; probably because the left ear was exposed to a higher intensity and continuous noise from the vehicle's window (27).

The results in table 4 showed the estimated hearing loss per years of exposure to noise. The maximum hearing loss was at 4000 Hz and after that at 8000 Hz. In this study, the age at employment variable and the interaction between age at employment and work experience was significant at 8000 Hz. Hearing loss in this frequency was related to age at employment. 

The negative interaction coefficient between work experience and initial hearing threshold means that the higher a person's initial hearing threshold baseline, the less is the hearing loss slope with aging. This decrease in the hearing threshold slope of workers with a higher initial hearing threshold is partly related to this fact that these workers are warned about their high hearing threshold at employment and are pursued to use protective and self-care equipment seriously. Thus, probably due to more self-care, their subsequent hearing loss is less than others. However, this effect may also be due to regression toward the mean.

The results of this study are in agreement with other studies, suggesting that noise-induced hearing loss mainly affects 4-8k Hz frequencies. Other studies have also shown occupational noise affects at 6kHz (28). Unfortunately, the data of this study did not include hearing loss at 6 kHz.

In a study conducted in 2014 on African gold miners, hearing loss at all frequencies in workers who were exposed to noise above 85dB, were higher in 3000 and 4000 Hz and higher in workers of 36 to 45 years of age (29). The current study also showed hearing loss was more severe at 4000 Hz, but unfortunately, no measurement at 3000 Hz was recorded.

Another study done on construction workers exposed to noise in Netherlands revealed 0.54 dB hearing loss per year in 3000, 4000, and 6000 Hz. In this study, particularly in the first decade of exposure to noise, there was a considerable increase in the hearing threshold (30). The current study implies that hearing loss is significantly associated with increased years of work which is in line with Loukzadeh et al.’ study in the tile industry (31). The results of a similar study also showed that with increase in years of work, hearing loss also increases (32).

In a study done by Cantley et al. on different levels of noise pollution at workplace and occupational hearing loss, it was shown that hearing loss caused by noise follows a dose-response pattern, i.e. people who were exposed to noise above 88 dB had a relative risk for hearing loss of 2.29, and those who were exposed to 85-88 dB noise had a relative risk of 1.39, and those who were exposed to noise between 82-84 dB had a relative risk of 1.26 (8). However, in the present study due to the characteristics of the workplace and the labor relocation in different parts, workers’ grouping according to exposure intensity was not done.

Although smoking is a known risk factor for many cancers and cardiovascular diseases, the relation between smoking and hearing loss is controversal. Faramarzi et al's study done on the effects of smoking, noise exposure, and aging on hearing loss showed that age and exposure to noise are independently effective on hearing loss. In their study smoking and age in people not exposed to noise, and smoking and noise exposure in people aged 20-40 years had an effect on hearing loss (22). The findings of this study also showed a significant negative interaction between smoking and work experience and a significant positive interaction between smoking and age at start of employment on hearing loss at some frequencies, but smoking alone had no significant effect on hearing loss. Palmer et al.’s study on occupational exposure to noise and hearing problems showed smoking-induced hearing loss in low frequencies (speech frequencies) (33). However, other studies showed smoking-induced hearing loss in high frequencies (34,35).

The study conducted by Banan et al showed no significant relation between smoking and hearing loss (28). In another study conducted by Starck et al, different risk factors such as smoking, systolic blood pressure, cholesterol level, and use of painkillers explained 36% of the hearing loss at 4000 Hz (36).

The results of the present study shows hearing loss in workers exposed to noise above 85 dB along with increase in their working years. Since noise-induced hearing loss can cause degeneration of the external cochlear hairy cells and considering the fact that there is no cure for this, preventing exposure to noise and identifying risk factors that may accelerate occupational hearing loss is crucial.

All workers are screened before starting their job at noisy factories and workers who already have serious hearing problems, and may cause liability for the factory are excluded. Therefore, for such a population the main factor causing hearing loss was more likely to be occupational noise. However, there might have been other factors that caused some hearing loss such as exposure to heavy metals (37), but in this study we did not have information about other factors and this was a limitation.

## Conclusion

This study shows that exposure to noise is still causing hearing loss in industries, despite the fact that workers and employers know the adverse effects of noise and have been instructed to use protective gear. Employers should put workers under more surveillence for using protective gear. Smoking should also be considered as a possible risk factor for hearing loss.
